# Prevalence of Early Childhood Caries in 3- to 6-Year-Old Children in Serbia: A National Pathfinder Study

**DOI:** 10.3390/children12060692

**Published:** 2025-05-28

**Authors:** Tamara Peric, Evgenija Markovic, Jovan Markovic, Bojan Petrovic, Biljana Kilibarda, Ana Vukovic, Dejan Markovic

**Affiliations:** 1 Clinic of Pediatric and Preventive Dentistry, School of Dental Medicine, University of Belgrade, 11000 Belgrade, Serbia; tamara.peric@stomf.bg.ac.rs (T.P.); ana.vukovic@stomf.bg.ac.rs (A.V.); dejan.markovic@stomf.bg.ac.rs (D.M.); 2 Clinic of Orthodontics, School of Dental Medicine, University of Belgrade, 11000 Belgrade, Serbia; jovan.markovic@stomf.bg.ac.rs; 3 Department of Pediatric and Preventive Dentistry, Dentistry Clinic of Vojvodina, Faculty of Medicine, University of Novi Sad, 21000 Novi Sad, Serbia; bojan.petrovic@mf.uns.ac.rs; 4 Institute of Public Health of Serbia “Dr. Milan Jovanovic Batut”, 11000 Belgrade, Serbia; kilibarda_b@batut.org.rs

**Keywords:** oral health, caries experience, preschool children, Serbia

## Abstract

This study aimed to assess dental caries status of 3- to 6-year-old preschool children in the Republic of Serbia. Stratified cluster sampling was implemented. Children were examined in four administrative regions of Serbia. Dental caries indices (decayed, missing, and filled) for deciduous teeth and the first permanent molar were recorded at the tooth level (dmft, DMFT) and surface level (dfs, DFS). In addition to cavitated caries lesions (ICDAS 3-6), visual changes in enamel (ICDAS 1-2) were recorded. This study included 1060 3-year-old and 1820 6-year-old children in Serbia. Fifty-six percent of 3-year-olds and 21% of 6-year-olds were caries-free. The mean dmft was 2.11 ± 3.45 for 3-year-olds and 4.46 ± 3.69 for 6-year-olds. The mean dfs was 2.62 ± 5.56 for 3-year-olds and 6.06 ± 6.33 for 6-year-olds. The decayed component was dominant in dmft/dfs. The prevalence of surfaces with initial lesion was 8% for 3-year-olds and 13% for 6-year-olds. Among children diagnosed with early childhood caries, 54% of 3-year-olds and 37% of 6-year-olds had a severe form. The mean DMFT was 0.15 ± 0.55, with only the first permanent molars being affected. Dental caries remains a significant public dental health issue among preschool children in Serbia. There is a noticeable trend of increasing numbers of decayed teeth as children progress through their preschool years. It is imperative to take corrective action enhance the existing oral health prevention program in Serbia with the aim of achieving better dental health among preschool children.

## 1. Introduction

Early childhood caries (ECC) is defined as the presence of one or more decayed, restored and missing teeth (due to caries), in any deciduous tooth in a preschool child up to six years of age [1]. It is a disease that can rapidly and irreversibly destroy deciduous teeth, initiating pain, infection, disturbed sleep, gastrointestinal disorders, and malocclusions [1]. Implementing preventive measures during early childhood, when deciduous teeth are erupting, is crucial for reducing the incidence of ECC. Therefore, ECC, the most frequently occurring chronic disease in children aged 2 to 6 years old according to the World Dental Federation (FDI), is considered a priority for preventive and treatment actions [2]. Establishing good oral health habits early on not only promotes healthier dentition but also enhances children’s quality of life.

However, the influence of early childhood caries on oral health and overall well-being has not been prioritized or addressed in Serbian society. This is evident even among dental professionals, whose participation in preventive oral health activities varies greatly. [3]. The latest report on the prevalence of ECC in Serbia was published in 2009, revealing that 55.4% of 3-year-old children and 21.7% of 6-year-olds had all healthy teeth [4].

As the process of urbanization and industrialization progressed in Serbia, so did sugar intake, and dietary customs have changed from traditional low-sugar diets to more westernized diets. Inequalities in family and regional income interact to create a caries profile in children across Serbia [4].

Children with caries in early childhood often continue to experience dental issues. Since the presence of cavities in the deciduous dentition is the strongest predictor of caries in permanent teeth, it is crucial to implement a strong caries prevention program [5]. In 2019, an initiative was introduced to update preventive oral health programs for children, intended to develop a more up-to-date program and shift the focus toward a more inclusive and preventive model for oral diseases management. It is essential to have knowledge of the distribution of caries in different regions of the country in order to recognize potentially more affected regions and consequently reinforce preventive and curative measures.

An epidemiological study on a large sample would generate evidence as a useful and powerful tool for creating reality-based public health policies and effective preventive caries programs. 

An enhanced program would improve the quality and availability of dental care, allowing more children to receive preventive care at an earlier age. This approach should ensure that preventive measures and dental services are available to all children, thereby reducing the incidence of cavities in the young population and minimizing unnecessary costly treatment.

The initial step in creating an effective contemporary preventive program for dental disease in young children is to collect epidemiological data on the prevalence of ECC through a national-level oral health survey that assesses the epidemiology at the population level. The null hypothesis in this study was that there would be no significant difference in the ECC profile among 3- to 6-year-old children across various living areas and administrative regions of Serbia. This paper aims to show the prevalence, severity, and pattern of ECC in 3- and 6-year-old children in the Republic of Serbia.

## 2. Materials and Methods

This study was part of a survey conducted at the national level, focused on assessing the oral health status of children and adolescents in Serbia. The investigation was conducted in accordance with the World health Organization (WHO) methodology for oral health surveys [6] approved by the Ethics Committee at the University of Belgrade School of Dental Medicine (document 36/10) and supported by the Ministry of Health of Republic of Serbia (document 500-01-49-3/2019-07).

According to the WHO guidelines [6], in populations with a high prevalence of caries (i.e., where less than 20% of 12-year-olds have all healthy teeth), the recommended sample size for each age group of children at every examination site is approximately 50 participants. The findings of a study conducted in Serbia in 2009 indicated that 18% of 12-year-olds were caries-free [4]. Consequently, the sample size for the oral health survey in Serbia was planned to involve examining 50 children aged 3 and 6 years old at various sample sites. These sites included four sites in the capital city (two urban and two peri-urban), six sites in three towns across three administrative areas (one urban and one peri-urban in each town), and one sampling site in each of the four rural regions. This ensured a minimum of 700 children in each age group.

The clinical examination was conducted at the primary health centers after written informed institutional consent was obtained. Thirty-three dentists from the public sector, who are trained to provide dental services for children, underwent the calibration process as previously described and participated in performing the clinical examination [7,8]. Briefly, calibration was conducted in two stages. Initially, three principal investigators were calibrated. Subsequently, thirty-three examiners participated in a training session. Each principal investigator supervised a group of 11 examiners. When diagnosing primary teeth caries, the overall Kappa was 0.95 for the principal investigators and 0.98 for all examiners.

Three-year old children were evaluated at the following sites: three urban and two peri-urban sites in the capital city of Belgrade, two urban, one peri-urban, and one rural site in Vojvodina, three urban, one peri-urban, and two rural sites in the Central and Western regions, and three urban, one peri-urban, and two rural sites in the Southern and Eastern regions. Six-year-old children were evaluated at four urban and three peri-urban sites in the capital city, four urban, one peri-urban, and four rural sites in Vojvodina, five urban, one peri-urban, and four rural sites in the Central and Western regions, and five urban, one peri-urban, and four rural sites in the Southern and Eastern regions. 

Children were scheduled for their annual dental check-ups through pediatric healthcare services in primary health care centers. Of the children scheduled for dental examination, the children included in the study were selected using a table of random numbers (50 children per site, along with a reserve group to account for absences due to illness or failure to attend the examination). However, in centers where the total number of scheduled children was approximately 50, all children were included in the sample. Prior to the clinical examination, written informed consent was obtained from the parents, and informed assent was obtained from the children.

Information on sociodemographic determinants, parents’ education levels, and access to primary health centers for dental prevention and treatment services was collected. Parental education level was recorded as follows: (1) college/faculty, (2) high school, or (3) elementary school.

Dental status examinations were conducted in ambulances at primary health centers between March and July 2019, with a dental mirror and probe. The dental status was recorded in the WHO Oral Health Assessment Form for Children, which was translated into Serbian. Dental caries indices (decayed, missing, and filled) were recorded for both primary and permanent dentition at the tooth (dmft, DMFT index) and surface levels (dfs, DFS index) [6]. Cavitated (ICDAS 3-6) and non-cavitated caries lesions (ICDAS 1-2) [9] were also documented. Severe early childhood caries (S-ECC) was defined in 3-year- old children as having one or more cavitated, missing (due to caries), or filled smooth surfaces in the deciduous maxillary frontal teeth. For 6-year-old children, a score of six or more decayed, missing, or filled teeth was indicative of S-ECC [10]. The severity of caries disease was presented as a Significant Caries Index (SiC Index), which was calculated as one-third of the mean values of the highest dmft score [11].

Data were prepared in Microsoft Excel (Microsoft Office Professional Plus 2021) and analyzed using SPSS 29.0 (IBM Corp. Released 2023. IBM SPSS Statistics for Windows, Version 29.0. Armonk, NY, USA: IBM Corp.). Parametric tests (the *t*-test or ANOVA) and non-parametric tests (the Mann–Whitney test or Kruskal–Wallis test) were used for the comparison. A significant difference was noted when the *p*-value was lower than 0.05.

## 3. Results

The study sample included 1060 3-year-old and 1820 6-year-old children living in the Republic of Serbia.

Fifty-six percent of 3-year-old children and 21% of 6-year-old children had all healthy teeth. Severe early childhood caries was diagnosed in 54% of 3-year-olds and 37% of 6-year-olds with ECC.

For 3-year-old children, the mean dmft index was 2.11 ± 3.45 (median 0), the number of decayed teeth was 1.93 ± 3.34, and the number of restored teeth was 0.17 ± 0.69. For 6-year-olds, increase in the dmft index (4.46 ± 3.69; median 4), the number of decayed teeth (3.53 ± 3.56), and the number of restored primary teeth (0.75 ± 1.55) were found. The mean dfs was 2.62 ± 5.56 (median 0) for 3-year-olds and 6.06 ± 6.33 (median 5) for 6-year-olds.

The most frequently affected surfaces with initial caries were occlusal (8% for 3-year-olds and 13% for 6-year-olds).

Primary tooth status for 3- and 6-year-old Serbian preschool children according to sociodemographic factors (gender, area, and administrative region) are shown in Table 1 and Table 2.

Among 3-year-olds, caries status was not significantly different with regards to gender, living area, or administrative region. However, 6-year-old children living in urban areas had lower rates of S-ECC compared to those in suburban and rural areas (*p* < 0.001, Kruskal–Wallis test). Additionally, a statistically significant difference was found in S-ECC between Central and Western Serbia and the city of Belgrade and Southern and Eastern Serbia (*p* < 0.05, Kruskal–Wallis test). Significantly lower dmft values were found in 6-year-olds residing in the capital city of Belgrade, and significantly higher dmft values were found in 6-year-olds residing in Southern and Eastern Serbia (*p* < 0.001, ANOVA, with Post Hoc Tests Multiple Comparisons).

The largest components of the dmft/dfs index were decayed teeth (d 89%, m 1%, f 10%; ds 89%, fs 11% for 3-year-olds, and d 76%, m 4%, f 20%; ds 78%, fs 21% for 6-year-old children). Three-year-olds in suburban areas had a significantly greater number of decayed teeth and a lower share of filled teeth compared to urban and rural areas (*p* < 0.05, ANOVA, post hoc test, Table 1). For 6-year-olds, there was a significant difference in the dmft composition between Central and Western Serbia and other regions (Mann–Whitney U test, Table 2).

The mean SiC dmft was 5.87 ± 3.74 (median 5) for 3-year-olds and 8.69 ± 2.48 (median 8) for 6-year-old children. The SiC dmft distribution is presented in Table 3. The SiC dmft was found to not be significantly different among 3-year-olds based on gender, area, or administrative region (*p* > 0.05, Kruskal–Wallis Test). For 6-year-old children living in the capital city of Belgrade, the mean SiC dmft was lower compared to those living in Central Western, Southern and Eastern Serbia (*p* < 0.001, Kruskal–Wallis Test).

Six-year-olds had an average of 4.45 ± 3.53 erupted permanent teeth. The mean DMFT was 0.15 ± 0.55, with only the first permanent molars being affected. The DMFT structure was D 80%, F 20%, with occlusal surfaces being the most affected. Of carious surfaces, 36% were with initial caries, and 64% had obvious cavities. There was a significant difference in the allocation of %D and %F component between the administrative areas of the country (Belgrade: D 88%, F 12%; Vojvodina: D 94%, F 6%; Central and Western Serbia: D 81%, F 19%; Southern and Eastern Serbia: D 66%, F 34%; *p* < 0.05, Kruskall–Wallis test).

Parental education levels did not influence the dmft values in 3-year-old children (*p* > 0.05, Kruskal–Wallis test). Six-year-old children whose mothers and fathers hold a university degree had significantly less caries compared to children whose parents finished high or primary school (*p* < 0.001, Kruskal–Wallis test). However, the d component was predominant with no significant difference among children with parents of various educational levels (*p* > 0.05, Kruskal–Wallis test).

Based on the frequency of dental visits per year, the observed dmft for children visiting a dental office once a year or two or more times a year showed no statistically significant differences in 3-year-olds (*p* > 0.05, Kruskal–Wallis test). Six-year-old children who have never visited dentist exhibited higher dmft, but without statistical significance when compared to those who have had one or more dental check-ups per year (*p* > 0.05, Kruskal–Wallis test).

## 4. Discussion

The present study was conducted under the umbrella of the WHO with the goal of collecting and presenting data on the dental health of 3- and 6-year-old children in Serbia. The sensitive transition from primary to mixed dentition is an important phase for the application of preventive caries measures, as early childhood presents a key opportunity for intervention. The results of investigating dental health trends in children as they grow are a powerful tool for examining the effectiveness of prevention programs and the functioning of the healthcare system.

Although the WHO recommends updating oral health data every five years by conducting national surveys [12], the last national study in Serbia was conducted in 2008. National oral health studies in Serbia from 2004 and 2008 have shown reduction in the number of 3-year-old children with all healthy teeth, dropping from 63.9% to 55.4%. The results of the present study showed a similar incidence of toddlers with healthy teeth (56%) compared to the findings from 2008 (55.4%). However, a worrying trend of a substantial decline in the number of children with all healthy teeth between the ages of 3 and 6 is still present. No significant changes in the number of caries-free 6-year-olds (21%) have been noted compared to the findings from 2004 and 2008 (18.6% and 21.7%, respectively) [4]. The decline in dental health from 2004 to 2008, followed by a slight improvement in over a 10-year period is concerning.

Despite the well-known measures for effectively preventing caries at an early age, ECC still persists as one of the most prevalent childhood diseases. Globally, discouraging data from a recent meta-analysis of the worldwide prevalence of ECC also showed no significant improvement over a 10-year period (1990–2019). ECC and its increasing prevalence pose a significant public health issue worldwide, impacting both developed and developing countries. The combined prevalence remains at 48–49%, with variations seen both between and within countries [13,14].

ECC in European countries presents an increased burden with an average prevalence of 42–46.2% [15,16,17,18,19,20]. Factors that largely contribute to the occurrence of early childhood caries include a high-sugar diet, parental socioeconomic status, a low level of parental education, and limited coverage and access to dental health services [21].

The WHO set a goal for 80% of children to have all healthy teeth by the year 2020 [22]. However, there is a large gap between the prevalence rate of ECC among Serbian preschool children and the anticipated goal. Considering the global rate of caries experience in young children, the set goal seems impossible to reach, especially in developing countries. The results of the present study indicate that the prevalence of ECC increases during childhood, which is expected due to the cumulative nature of the disease. Clinical prevention and treatment services for children are limited in accessibility and not necessarily free of charge in most countries. Early childhood caries is an etiologically complex disease that develops as a result of multiple risk factors, as well as and favorable dietary, socio-economic, behavioral and environmental influences [23,24,25]. These factors might affect the delivery of dental care services and the successful realization of preventive programs.

A positive association of ECC with socioeconomic indicators such as geographical area and gross national income is noted in the majority of studies [20,26,27]. Geographical area and family income are factors influencing dental health in Serbia. Children from lower-income families, mostly residing in rural regions of Serbia, have a high prevalence of caries. This is consistent with findings in Italy, as well as across Europe [17,18,19]. A Swedish multilevel analysis showed that children whose parents have low socioeconomic positions and education levels are at a higher risk for developing ECC [28]. It has also been observed that knowledge and attitudes about dental health, as well as the provision of dental services, vary among administrative regions. This is consistent with the findings of similar studies conducted in other regions of Europe [20,26,28].

The higher prevalence of S-ECC in 3- and 6-year-olds was found in rural areas compared to urban regions in Serbia. Preschoolers living in the city showed significantly lower S-ECC rates compared to children from rural and suburban sites, which is consistent with the results of a recent epidemiological investigation from the region [17,18,19,20].

According to the WHO caries severity criteria [6], Serbian preschoolers are classified in the high-caries-risk group, exhibiting similar dmft values compared to high-risk countries in the Eastern European region [17,29,30,31], but a higher dmft index compared to low-caries-risk, high-income countries in the European region. Previously reported dmft rates vary from 11% to 23% in high-income countries [27,32,33,34,35,36,37,38]. 

The WHO SiC Index target for 12-year-olds is less than 3.8 [11]. The SiC Index was intended to focus on children with the highest DMFT/dmft scores in order to bring about changes in preventive policies. Unfortunately, not many epidemiological studies on the prevalence of caries in children with primary dentition have calculated the SiC Index. Currently, there is no WHO target for SiC levels in deciduous teeth. The epidemiological studies on the prevalence of caries in primary dentition are scarce, especially those presenting the SiC Index. Serbian children aged 3 to 6 years have a higher SiC Index compared to the results of a recently published study on children in Germany [39], but lower compared to a small sample of children from the USA [40]. The SiC Index is more accurate in assessing caries morbidity than dmft and is very useful tool for identifying the most affected group of children with the highest dmft scores. High scores highlight the need for better preventive programs in the very young population. A target score from the WHO would promote a more standardized approach to epidemiological studies in dentistry and community health-related studies. 

The current trend in high-income countries is a costly, treatment-oriented specialist approach, coupled with widely spread preventative measures. However, there is a discrepancy between available dental care services and their full dissemination and application among different social and ethnic populations. This leads to disparities in the delivery of dental services and results in uneven dental health statuses. In countries with low- and middle-income economies, dental services are often lacking in quantity and quality in public institutions and are unaffordable in the private sector for the majority of the population, especially for children living in rural regions [32,33,34,35,36,37,38,41,42].

The reported percentage of untreated ECC in 3- to 6-year-old children living in Serbia is high, exceeding the European average [13,14]. More than half of the 3-year-old children with caries included in the present study had S-ECC. Most cavities in 3- and 6-year-old Serbian children remained untreated, with occlusal surfaces being the most commonly affected, as found in a study conducted in England [43]. The significant difference in the percentages of decayed and filled tooth surfaces between urban rural regions in Serbia could be attributed to the lower-income economy and availability of dental services. As the capital city, Belgrade is the most developed socioeconomic center in the country [44], with the highest number of children with all healthy teeth.

Another very important reason for the high number of caries-affected teeth compared to restored teeth may be the lack pediatric dentists in the public dental sector. According to the 2019 annual reports, 1,276,654 children aged 0–18 years reside in Serbia [45]. The number of dentists involved in working with children in public health institutions is only 322 [46]. This means that each dentist provides dental treatment and preventative services for an average of almost 4000 children, which is in conflict with good practice recommendations (one pediatric dentist should provide dental services for a maximum of 1500 children) [47]. The limitations of the national dental healthcare system play a crucial role in the quantity and quality of dental services that are delivered. Additionally, fixed wages and a large number of patients per dental practitioner may result in insufficient provision of treatment, as seen in other European countries facing similar situations [41,42,43,44,45,46,47,48]. One more limiting factor in providing adequate dental care could be constraints connected to the provision of dental care to young children, which is time-consuming and influenced by the nature and compliance of the child. Thus, the rate of the provision of dental services to young children cannot be equalized with the same kind of dental service provided for the schoolchildren or adolescents because of the challenges of working with toddlers and preschoolers. A similar insufficient provision of dental services in 3-year-old children was found in Germany and presented in a recent study [39].

The educational levels of both parents had a significant impact on the dmft index, as previously reported [28]. The findings of the present study show a difference in the dmft index among 6-year-olds based on their parents’ levels of education, but not among 3-year-olds. Interestingly, despite varying socioeconomic backgrounds among parents, the d component of the dmft index was nearly identical in children with highly educated parents compared to children with parents who had less education. The significant number of decayed teeth among children regardless of their parents’ education levels, along with the low number of restored teeth, indicates deficiencies and shortcomings in the healthcare system rather than a lack of parental understanding about caries prevention. It seems that the main reason for most visits to the dental office is for the treatment of existing carious lesions and emergency care, rather than for prophylaxis or check-ups. These findings align with the results reported by Kilibarda et al. [3]. The correlation between the frequency of dental visits per year and the dmft shows similar values regardless of the number of visits to pediatric dentists, with a dominant d component. This information once again highlights the insufficient number of dentists working with children, who are unable to manage the high volume of patients in need, as previously reported [3,42,48].

Historically, dental healthcare has been a low priority for the public health system in Serbia, a trend that is also common in the majority of developing countries worldwide [49]. Funding for the public dental sector, individual dental coverage, and the allocation of dentists providing dental services in primary healthcare institutions are important factors in reducing the prevalence of ECC [42,50]. Higher government expenditure on dental health may be associated with a decrease in the rate of ECC [51], but the tough economic situation in the country has not allowed for much spending in the area of dental health. Economic factors have a significant impact on dental healthcare policies in Serbia, leading to a noticeable lack of availability of dental services. Since dental treatment and preventive services are funded by the government, the current caries prevention program, particularly for early childhood, needs additional attention. The current prevention and treatment measures do not adequately address the dental health needs of children. The necessary corrective actions to reform the existing dental health prevention program in Serbia are essential in order to implement strategies for reducing ECC.

The strengths and limitations of this study must be taken into consideration. The large sample size provided appropriate reliability and ensured that the findings are a true representation of the population’s characteristics. The children enrolled in the study were from the general population in Serbia. The number of dental visits and the need for dental services were not factors that influenced the recruitment process. The study was conducted by pediatric dentists who are trained and calibrated to ensure the standardization of the examinations performed. Standardized survey techniques and methods produced reliable information. One of the study limitations was that the rate of failing restorations was not considered. These data could provide valuable information on the quality of dental work, which may be affected by time constraints, challenges in working with young children, or local factors such as diet. Additionally, there may be a limitation in assessing incipient interproximal caries in toddlers, which could have been influenced by obstructed visual detection due to adjacent restorations or caries, potentially leading to an underestimation of the caries status. The authors of the present study encountered limitations when analyzing responses to the parental-reported dietary and oral hygiene questionnaire. These limitations deter the inclusion of the findings in the results due to the potential for errors resulting from forgetfulness or parents’ reluctance to admit to unhealthy behaviors. These responses could have introduced response bias or social desirability bias and, therefore, were omitted. 

Despite the acknowledged limitations, the findings of this study offer important information about dental status and the prevalence of ECC in 3- to 6-year-old children in Serbia. Since the recent initiative aimed at improving preventive policies for ECC, the findings can be utilized to update the current preventive dental care program.

## 5. Conclusions

Early childhood caries is a significant public health issue among 3- to 6-year-old children in Serbia. The findings of the present study partially rejected the null hypothesis: A significantly lower caries experience was found for 6-year-old children living in the capital city of Belgrade compared to preschoolers living in other administrative regions of Serbia. However, no differences were found in the group of 3-year-olds. Only half of Serbian 3-year-old children and around one-fourth of 6-year-old children have healthy teeth. Of all children diagnosed with ECC, more than half of toddlers have the severe form. A considerably high rate of S-ECC was found in 6-year-old preschoolers. Preschool children in rural regions were more affected compared to those living in urban environments. Serbian children aged 3 to 6 years belong to the high-caries-risk group. All children affected with caries had the highest number of unrestored teeth compared to those that were restored or missing. The findings form this study lay the groundwork for further research on caries severity, morbidity, and control, as well as strategies to reduce the costly interventions needed for treating caries.

## Figures and Tables

**Table 1:** Prevalence of 3-year-old children with all healthy teeth, S-ECC [%], dmft, dfs, and dmft structure.

	Number of Children	Caries-Free %	S-ECC %	dmft Mean ± SD (Median)	dfs Mean ± SD (Median)	dmft Structure		
						%d	%m	%f
Gender								
Boys	550	58%	54%	2.04 ± 3.40 (0)	2.56 ± 5.09 (0)	90.4%	0.3%	9.3%
Girls	510	54%	53%	2.18 ± 3.51 (0)	2.67 ± 5.99 (0)	88.5%	0.9%	10.5%
Area								
Urban	560	54%	50%	2.09 ± 3.36 (0)	2.59 ± 5.58 (0)	88.1%	0.8%	11.1% ^a^
Suburban	250	58%	62%	2.26 ± 3.71 (0)	2.92 ± 5.85 (0)	96.9%	0.2%	2.9% ^a,b^
Rural	250	60%	58%	2.03 ± 3.49 (0)	2.41 ± 5.16 (0)	87.1%	0.2%	12.7% ^b^
Administrative regions of Serbia								
Belgrade	250	54%	52%	2.06 ± 3.32 (0)	2.35 ± 4.56 (0)	92.2%	0.1%	7.7%
Vojvodina	200	64%	55%	1.90 ± 3.59 (0)	2.37 ± 5.03 (0)	85.3%	0.9%	13.8%
Central and Western Serbia	305	53%	55%	2.32 ± 3.66 (0)	3.05 ± 6.98 (0)	91.6%	0.6%	7.8%
Southern and Eastern Serbia	305	56%	53%	2.02 ± 3.22 (0)	2.42 ± 4.37 (0)	87.1%	0.7%	12.2%

^a, b^ Significant differences between values are indicated with identical letters (*p* < 0.05, ANOVA, post hoc test).

**Table 2:** Prevalence of 6-year-old children with all healthy teeth, S-ECC [%], dmft, dfs, and dmft structure.

	Number of Children	Caries-Free %	S-ECC %	dmft Mean ± SD (Median)	dfs Mean ± SD (Median)	dmft Structure		
						%d	%m	%f
Gender								
Boys	960	20%	36%	4.54 ± 3.73 (4)	5.79 ± 5.67 (5)	76.5%	19.8%	3.7%
Girls	860	21%	38%	4.38 ± 3.65 (4)	6.37 ± 6.99 (5)	76.0%	19.8%	4.2%
Area								
Urban	920	20%	31% ^a,b^	4.36 ± 3.60 (4)	5.36 ± 5.85 (5)	76.3%	4.2%	19.5%
Suburban	300	22%	45% ^a^	4.73 ± 3.90 (4)	6.30 ± 4.97 (4)	76.5%	3.5%	20.0%
Rural	600	24%	44% ^b^	4.48 ± 3.76 (4)	5.77 ± 5.52 (4)	87.1%	3.7%	20.2%
Administrative regions of Serbia								
Belgrade	370	25%	33% ^c^	3.76 ± 3.46 (3) ^A,B,C^	5.48 ± 5.85 (4)	77.4%	5.1%	17.5% ^e^
Vojvodina	450	19%	37%	4.39 ± 3.62 (4) ^A,D^	6.23 ± 6.44 (5)	76.2%	3.2%	20.6% ^f^
Central and Western Serbia	500	22%	42% ^c,d^	4.41 ± 3.69 (4) ^B,E^	6.18 ± 5.59 (4)	72.1%	3.8%	24.1% ^e,f,g^
Southern and Eastern Serbia	500	19%	34% ^d^	4.93 ± 3.81 (5) ^C,D,E^	6.06 ± 7.15 (6)	80.2%	4.2%	15.6% ^g^

^a, b^ *p* < 0.001, Mann–Whitney U test between the values marked with the same letter; ^c, d, e, f, g^ *p* < 0.05, Mann–Whitney U test between the values marked with the same letter; ^A, B, C, D, E^ *p* < 0.001, ANOVA, post hoc tests multiple comparisons.

**Table 3:** The SiC dmft distribution.

	3-Year-Olds Mean ± SD (Median)	6-Year-Olds Mean ± SD (Median)
Gender		
Boys	5.74 ± 3.70 (4.5)	8.77 ± 2.58 (8)
Girls	6.00 ± 3.80 (5)	8.59 ± 2.36 (8)
Area		
Urban	5.82 ± 3.69 (5)	8.55 ± 2.53 (8)
Suburban	6.08 ± 3.88 (4)	8.99 ± 2.54 (8)
Rural	5.86 ± 3.83 (5)	8.75 ± 2.36 (8)
Administrative regions of Serbia		
Belgrade	5.70 ± 3.55 (4)	7.86 ± 2.32 (7) ^a,b^
Vojvodina	5.66 ± 4.23 (4)	8.52 ± 2.57 (8) ^c^
Central and Western Serbia	6.37 ± 3.84 (5)	8.63 ± 2.50 (8) ^a,d^
Southern and Eastern Serbia	5.52 ± 3.46 (5)	9.22 ± 2.43 (8) ^b,c,d^

^a, d^ *p* < 0.05, Mann-Whitney U test between the values marked with the same letter; ^b, c^ *p* < 0.001, Mann-Whitney U test between the values marked with the same letter.

## Data Availability

Datasets generated and/or analyzed during the study are available from the first author on reasonable request.
